# A complete reference genome reveals genetic landscape of *Brassica* centromeres

**DOI:** 10.1186/s13059-026-04218-4

**Published:** 2026-07-27

**Authors:** Xin-Dong Xu, Si-Ying Ye, Pei Wu, Yong-Qing Qian, Zu-Wen Zhou, Yibo Chai, Ziyan Feng, Yingying Tan, Jie Xiang, Lijuan Wei, Rui Wang, Qingyuan Zhou, Cunmin Qu, Jiana Li, Ling-Ling Chen, Kun Lu, Jia-Ming Song

**Affiliations:** 1 College of Agronomy and Biotechnology, Southwest University, Chongqing, 400715 China; 2 Yazhouwan National Laboratory, Sanya, 572025 China; 3 College of Life Science and Technology, Guangxi University, Nanning, 530004 China; 4 Engineering Research Center of Grain and Oil Crops in Southwest Mountain, Ministry of Education, Chongqing, China; 5 Key Laboratory of Germplasm Innovation of Upper Yangtze River, Ministry of Agriculture and Rural Affairs, Southwest University, Chongqing, 400715 China

**Keywords:** *Brassica napus*, Reference genome, Centromere evolution, Polyploidization

## Abstract

Centromeres are essential for chromosome segregation, yet their organization and evolution remain poorly understood in polyploid crops. We present the first complete telomere-to-telomere reference genome of *Brassica napus* cultivar Zhongshuang11 and characterize centromere architecture and evolution. We identify core satellite repeats that define centromeres and reveal extensive sequence polymorphism and structural divergence between subgenomes associated with polyploidization and domestication. We further uncover rDNA-enriched pericentromeres generated by recurrent chromosome rearrangements and propose a centromere evolutionary cycle model that explains how satellite repeats and transposons jointly shape centromere evolution.

**Supplementary Information:**

The online version contains supplementary material available at https://doi.org/10.1186/s13059-026-04218-4.

## Background

Centromeres are essential chromosomal regions required for accurate chromosome segregation and genome stability in eukaryotes. Over evolutionary time, centromeres undergo extensive turnover, including sequence replacement, expansion, contraction, and repositioning, profoundly shaping genome architecture and karyotype evolution [1, 2]. In most eukaryotes, centromeres consist of large arrays of tandemly repeated satellite DNA interspersed with transposable elements, forming structures dominated by satellite repeats, transposable elements, or a combination of both [3, 4]. Despite striking diversity in sequence composition and size across species, centromere function remains highly conserved, a phenomenon known as the centromere paradox [5, 6]. Owing to their extreme repetitiveness and complex higher-order organization, centromeres have long remained among the least resolved regions of eukaryotic genomes, limiting accurate genome assembly and functional characterization [6–9].

Recent advances in long-read sequencing technologies have begun to overcome these limitations. Telomere-to-telomere (T2T) genome assemblies in model plants such as *Arabidopsis* [6, 10], rice (*Oryza sativa*) [11], and maize (*Zea mays*) [12] have provided important insights into centromere structure and evolutionary dynamics. However, centromeres in allopolyploid crops formed through interspecific hybridization followed by whole-genome duplication remain poorly understood, as their duplicated and highly repetitive genomes pose substantial challenges for assembly and comparative analysis.

Polyploidization has played a central role in the evolution, adaptation, and agricultural success of many major crops [13–15]. Species of the genus *Brassica*, with their recent and well-defined polyploidization history, provide an excellent system for investigating centromere evolution [15, 16]. The allotetraploid *B. napus*, formed by hybridization between *B. rapa* and *B. oleracea*, is one of the world’s most important oilseed crops. In recent years, the first gap-free genome of the *B. napus* line Xiang5A (X5A) has been reported, resolving all remaining chromosomal gaps and uncovering previously hidden structural variations [17]. In parallel, a comprehensive pan-centromere landscape has been established across diverse *Brassica* germplasms, revealing remarkable intra- and interspecific centromeric diversity within the genus [18, 19]. Despite these advances, a T2T genome assembly of *B. napus* is still lacking, and a systematic dissection of centromere structural dynamics and their evolutionary trajectories remains incomplete. Previous chromosome-level assemblies of the elite cultivar Zhongshuang 11 (ZS11) have facilitated studies of subgenome differentiation and structural variation [20–22], but its resolution remains insufficient to resolve complex centromeric regions. In this study, we generate a T2T genome assembly of *B. napus* ZS11 and leverage comparative genomics across the *Brassica* lineage to dissect centromere evolution following polyploidization.

## Results and discussion

**Fig. 1:**
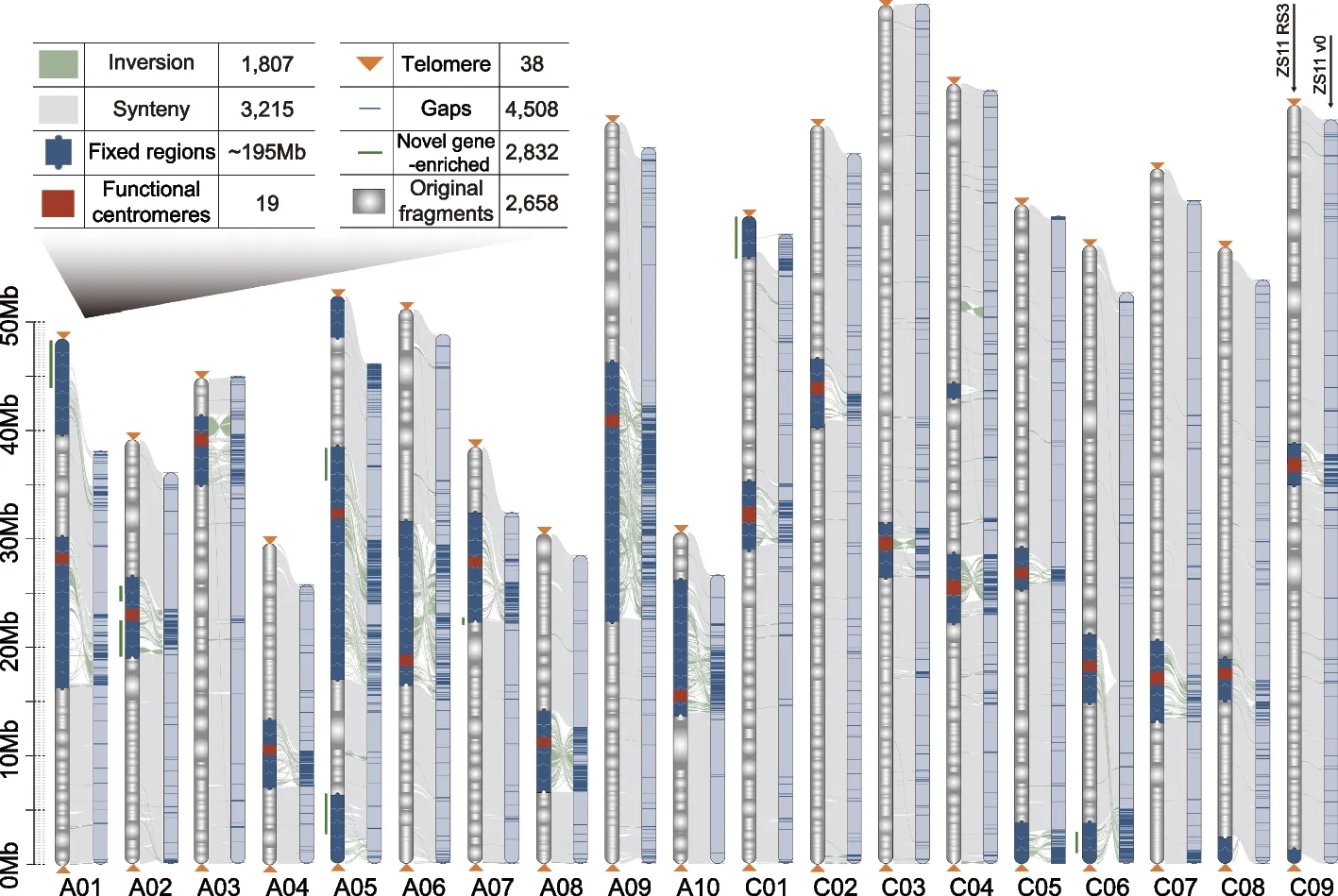
The final piece of the *B. napus* reference genome puzzle: complete centromere and telomere resolution. Synteny between the ZS11RS3 (first chromatid) and ZS11 v0 (second chromatid) assemblies. A01-10 denotes chromosomes derived from *Brassica rapa* while C01-C09 denotes chromosomes derived from *Brassica oleracea*. Blue segments on ZS11RS3 indicate regions repaired in the new assembly. Gradient-colored blocks along chromosomes denote syntenic regions between the two versions. Blue marks on ZS11 v0 indicate the positions of assembly gaps. The panel in the upper left summarizes key assembly metrics for both genome versions

By integrating 126.42 Gb PacBio reads (~ 125 ×) and 235.51 Gb Oxford Nanopore Technologies (ONT) reads (~ 235 ×), including 23.24 Gb of ultra-long reads, we generated a telomere-to-telomere (T2T), chromosome-scale genome assembly of the *B. napus* cultivar Zhongshuang 11 (ZS11), designated ZS11 Reference Sequence v3 (ZS11RS3). Compared with the previous ZS11 v0 assembly [22], contig N50 increased from 2.7 Mb to 56.26 Mb, all 4,508 gaps were closed, and telomeric repeats were detected at both ends of all 19 chromosomes (Fig. 1), establishing the first fully resolved *B. napus* genome. Repaired regions spanned 172.77 Mb, including 87.53 Mb of previously unassembled regions (PURs) [23] (Additional file 1: Fig. S1 and Additional file 2: Table S1), which were predominantly located in centromeric and pericentromeric regions. Genome completeness reached 100% based on BUSCO analysis (Additional file 2: Table S2), and k-mer-based quality assessment yielded a consensus QV of 71.48, corresponding to a base accuracy of 99.99999%.

Comparative analysis between ZS11RS3 and ZS11 v0 identified 3,215 syntenic blocks and 1,807 inversion blocks, approximately 90% of which were located within centromeric regions, underscoring the previous inaccessibility of these regions. The improved assembly enabled the annotation of 18,470 additional protein-coding genes, including 2,832 genes absent from ZS11 v0, many of which were clustered in formerly unresolved regions (Fig. 1). These improvements provide a complete chromosomal framework for resolving centromeric sequence composition and structure in an allopolyploid crop genome. To further evaluate the accuracy and structural integrity of the assembly, we performed a synteny analysis between the ZS11RS3 genome and the gap-free *B. napus* X5A genome [17]. The results revealed a high degree of collinearity between the two genomes, providing additional support for the reliability of our assembly (Additional file 1: Fig. S2).

**Fig. 2:**
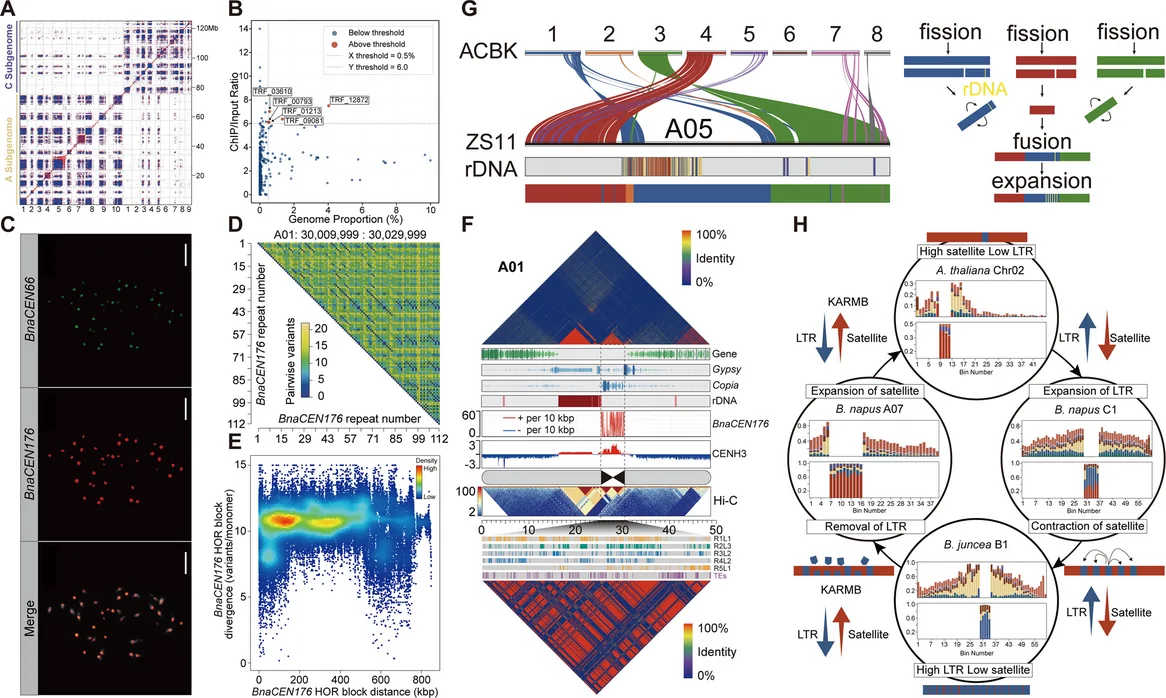
Decoding the final piece in *Brassica*: CDRs annotation, discovery of a novel rDNA-domain, and the evolutionary cycle. **A** Dot plots showing pairwise sequence similarity among the 19 centromeres based on a 176-bp window. forward and reverse strand matches are shown in red and blue, respectively. **B** Genome-wide distribution of repeat clusters in *B. napus* ZS11RS3 and their corresponding CENH3 ChIP/input enrichment ratios. **C** FISH localization of the centromeric satellites *BnaCEN176* (red) and *BnaCEN66* (green) on mitotic metaphase chromosomes of *B. napus* ZS11RS3. **D** Heatmap of pairwise sequence variation among *BnaCEN176* repeats within a representative region of centromere A01. **E** Relationship between inter-HOR block distance and sequence divergence among *BnaCEN176* HOR blocks within the chromosome A01 centromere dip region. Sequence divergence was quantified as the average number of variant sites per *BnaCEN176* monomer. **F** Multilayered structural organization of the A01 centromere, including gene density, LTR/*Gypsy* and LTR/*Copia* density, rDNA distribution, *BnaCEN176* copy number (10-kb windows; forward and reverse strands shown in red and blue), CENH3 enrichment (log₂ ChIP/Input), chromosome schematic, Hi-C contact map with TADs, HOR distribution, TE composition, and sequence identity. **G** Karyotype evolution of chromosome A05 from the ancestral ACBK configuration and the formation of REP regions, showing synteny, rDNA distribution, inferred ancestral blocks, and a schematic model of REP origin. **H** A genus-wide model illustrating cyclical centromere evolution in *Brassica*, driven by antagonistic interactions between satellite sequence homogenization (red) and retrotransposon invasion (blue)

Analysis of tandem repeats revealed that *BnaCEN176*, a 176-bp satellite monomer, represents the most abundant centromeric repeat in *B. napus*, with 364,330 copies detected in ZS11RS3 compared with only 107,514 in ZS11 v0 (Additional file 1: Figs. S3, 4). Genome-wide sequence analysis uncovered substantial monomer polymorphism, with clear differentiation between A- and C-subgenomes and extensive intra-chromosomal variation (Fig. 2A). Certain positions within the monomer exhibited elevated mutation frequencies (Additional file 1: Fig. S3). Based on the positional distribution of CENH3 ChIP-seq peaks, we further delineated the boundaries of the centromere dip regions (CDRs) [18, 24], which span 0.79–1.36 Mb. This region has also been referred to as functional centromeric chromatin in recent studies [25] (Additional file 1: Fig. S5 and Additional file 2: Tables S3, 4). Centromeric monomer libraries showed pronounced chromosome specificity in the A-subgenome, whereas centromeres in the C-subgenome shared more monomer copies (Additional file 1: Fig. S5). In addition to *BnaCEN176*, five previously uncharacterized satellite monomers enriched in CENH3-associated chromatin were identified (Fig. 2B, Additional file 1: Figs. S6, 7 and Additional file 2: Table S5), and fluorescence in situ hybridization confirmed centromeric localization of *BnaCEN176* and *BnaCEN66* (Fig. 2C).

As in other eukaryotes, centromeric satellite monomers in *B. napus* are organized into higher-order repeats (HORs), with 52,833 HORs detected across all CDRs (Fig. 2D and Additional file 1: Fig. S8). Although sequence divergence, measured as the average number of variant sites per monomer, generally increased with genomic distance between HOR blocks in *Arabidopsis* [10], this pattern was disrupted in several centromeres (Fig. 2E and Additional file 1: Fig. S8). For example, in chromosome A01, distant HOR blocks exhibited unexpectedly low divergence, consistent with historical physical proximity. Detailed sequence inspection revealed extensive transposable element (TE) insertions between these blocks, producing alternating satellite-rich and TE-rich segments (Additional file 1: Fig. S9). These findings suggest that TE insertions have contributed to the fragmentation of ancestral centromeric architectures in *B. napus*. Notably, the CDR of chromosome C04 lacked detectable satellite repeats and was dominated by TEs, indicating that TE-rich centromeres can independently recruit CENH3.

Integration of CENH3 enrichment profiles, sequence identity heatmaps, and satellite arrays delineated centromeric domains spanning 3.87–10.44 Mb in the A-subgenome and 3.72–7.16 Mb in the C-subgenome (Additional file 2: Table S6). Despite larger chromosome sizes, C-subgenome centromeres were significantly shorter than those of the A-subgenome (*P* < 0.05; Additional file 1: Fig. S10). Analysis of previously published Hi-C data from the ZS11 v0 genome, generated from the same biological material as used in this study [22], revealed that centromeres form compact three-dimensional chromatin domains with elevated interaction frequencies (Fig. 2F and Additional file 1: Figs. S11, 12). Although only ~ 2% of topologically associating domains (TADs) overlapped centromeres, centromeric TADs were significantly larger than those on chromosome arms (*P* < 0.0001) and were closely aligned with centromere boundaries (Additional file 1: Fig. S13, Additional file 2: Tables S7, 8), indicating spatial insulation of centromeric chromatin.

Unexpectedly, several chromosomes contained rDNA-enriched pericentromeric regions (REPs) that shared TADs with centromeres but lacked canonical centromeric satellites (Fig. 2F and Additional file 1: Figs. S11, 12, Additional file 2: Tables S9, 10). These regions showed significantly higher CENH3 occupancy than chromosome arms yet markedly weaker signals than centromeres (Additional file 1: Fig. S14). Comparative analysis across *Brassica* species revealed that REP regions are confined to the A-subgenome and predate the origin of *B. rapa* (Additional file 1: Fig. S15). Reconstruction of ancestral karyotypes indicated that REP regions correspond to ancestral chromosome fission–fusion boundaries, suggesting that terminal rDNA loci were relocated to internal positions during karyotype evolution and subsequently expanded locally (Fig. 2G and Additional file 1: Fig. S16, Additional file 2: Table S11).

To elucidate the forces driving centromere divergence, we compared satellite and TE dynamics before and after polyploidization. LTR retrotransposons dominated centromeric TEs, with LTR/*Copia* elements, preferentially enriched within centromeres, while LTR/*Gypsy* elements accumulated mainly in pericentromeric flanks (Additional file 1: Figs. S17-19). This lineage-specific compartmentalization was conserved across subgenomes and progenitors, indicating long-standing constraints on LTR integration (Additional file 1: Figs. S20, 21).

Insertion-time analysis showed that centromeric LTRs are significantly younger than those on chromosome arms, with more recent and pronounced LTR bursts in the A-subgenome (Additional file 1: Figs. S10, 22). To quantify LTR turnover, we calculated solo-to-intact LTR ratios as a proxy for removal efficiency. A-subgenome centromeres exhibited significantly higher LTR removal efficiency than those of the C-subgenome, resulting in retention of predominantly young LTRs, whereas inefficient removal in the C-subgenome allowed accumulation of ancient insertions (Additional file 1: Fig. S15). Extension of this analysis across *Brassica* species revealed consistent subgenome-specific patterns, with overall LTR removal efficiency following the order B > A > C (Additional file 1: Fig. S15, Additional file 2: Tables S12, 13).

Across *Brassica* genomes, centromeric LTR-to-satellite ratios varied widely, contrasting with the satellite-dominated centromeres of *Arabidopsis* (Additional file 1: Figs. S23, 24). These observations support a cyclic model of centromere evolution in *Brassica* (Fig. 2H), in which alternating phases of LTR amplification and satellite re-expansion are governed by lineage- and subgenome-specific LTR removal efficiency. Polyploidization reshapes this balance, driving rapid divergence of centromere length and composition while preserving centromere function.

## Conclusions

In this study, we present the first telomere-to-telomere genome assembly of *B. napus* cultivar ZS11 and use it to achieve a comprehensive dissection of centromere architecture, 3D organization, and evolutionary dynamics in an allopolyploid crop genome. By precisely defining centromeric and pericentromeric regions, we uncover pronounced divergence between subgenomes in sequence composition, chromatin organization, and transposable element dynamics following polyploidization. The discovery of rDNA-enriched pericentromeres, which originated from ancestral chromosome fission–fusion events, reveals an unrecognized layer of centromere-associated genomic complexity in *Brassica*. Most importantly, comparative analyses across multiple *Brassica* species demonstrate that differential long terminal repeat retrotransposon turnover, rather than satellite repeat expansion, is the primary driver of centromere diversification. These findings support a cyclic model of centromere evolution governed by the antagonistic interplay between LTR retrotransposons and satellite repeats, modulated by subgenome-specific removal efficiency. Together, our work provides a unified framework for understanding how centromeres evolve, diversify, and stabilize in polyploid plant genomes, with broad implications for chromosome biology, karyotype evolution, and genome stability under domestication.

## Methods

### Genome assembly and evaluation

The ZS11RS3 genome was assembled using Hifiasm (v0.25.0-r726) [26] with PacBio HiFi, ONT, and ONT ultra-long (UL) sequencing data, using parameters “--ul -t72”. Hifiasm was also employed to generate an additional assembly based solely on ONT UL reads with parameters “-o ZS11RS3 --ont -t72”. A third assembly version was produced using NextDenovo (v2.5.2) [27] based on ONT and ONT UL reads, with parameters set to “read_type = ont, read_cutoff = 1 k, genome_size = 1.2 g, sort_options = -m 50 g -t 35, pa_correction = 6, nextgraph_options = -a 1”. All three assembly versions were scaffolded against the ZS11 v0 reference using RagTag (v2.1.0) [28] with default parameters. Syntenic relationships among the assemblies were established using MUMmer (v4.0.1) [29] with parameters “-g 1000 -c 90 -l 40 -t 20”, and genomic gaps were subsequently filled based on these syntenic alignments. Preliminary assembly statistics, including contig N50 as a measure of genome contiguity, were calculated using SeqKit (v2.10.1) [30] with parameters “stat –all”. The completeness of the gap-filled assembly was evaluated using Compleasm (v0.2.7) [31], with parameters “--autolineage -t72”, while genome accuracy based on 13-mer frequencies was assessed using Merqury (v1.3) [32] with default settings. PacBio and ONT reads were mapped to the assembled genome using Winnowmap (v2.03) [33] with parameters “-W repetitive_k15.txt -ax map-ont” and “-W repetitive_k15.txt -ax map-pb”, respectively. SAMtools (v1.14) [34] was used for sorting and calculating coverage statistics. Previously unassembled regions (PURs) were identified by aligning ZS11RS3 to the ZS11 v0 reference genome. Regions present in ZS11RS3 but absent from ZS11 v0 were defined as PURs, following previously published criteria [23]. Genome-wide synteny between the ZS11 v0 and ZS11RS3 genome assemblies was visualized using GenomeSyn with default parameters [35, 36].

### Genome annotation

Transposable elements (TEs) in the ZS11RS3 genome were annotated using Extensive de-novo TE Annotator (EDTA) (v1.9.6) [37] with parameters “--anno 1 --threads 72”. To ensure consistency with the widely used ZS11 v0 reference, gene models were transferred from ZS11 v0 to ZS11RS3 using LiftOn (v1.0.5) [38] with default parameters, while preserving the original gene IDs. De novo gene prediction on the assembled genome was conducted using Helixer (v0.3.6) [39] with parameters “--lineage land_plant”. Ribosomal DNA (rDNA) regions were annotated using Barrnap (https://github.com/tseemann/barrnap) with parameters “--kingdom euk --threads 72”.

### Centromeric satellite annotation

Centromeric satellite sequences were identified based on tandem repeats using TRASH [40] with default parameters. Repeat dot plots were generated using ModDotPlot (v0.9.8) [41] with parameters “--color #132D88 #6E4698 #459,567 #EFEA3A #E73119 -id 0 -w 10,000”. Comparative analysis of centromeric sequences in 176-bp windows was conducted with reDOTable (v1.2) (https://github.com/s-andrews/redotable). To identify satellites enriched in centromeric regions from CENH3 ChIP-seq data, we adopted a strategy similar to that used in cotton genome studies [42]. Tandem repeats were first identified using TRF (v4.09.1) [43] with parameters “2 7 7 80 10 50 500 -f -d -m”, and subsequently clustered using MMseqs2 (v18-8cc5c) [44] with parameters “easy-cluster clusterRes tmp --min-seq-id 0.5 -c 0.8 --cov-mode 1”. ChIP and input reads were then aligned to the clustered repeats using BLASTn (v2.16.0) [45]. Normalized ChIP/Input values were then calculated for each repeat to quantify enrichment.

### Probe labeling and fluorescencein situhybridization (FISH)

*BnaCEN176* and *BnaCEN66* sequences were directly synthesized, and cloned into a PMD19-T vector. Subsequently, the plasmids were extracted and subjected to enzymatic digestion using the Nick Translation System kit (Invitrogen, USA) for labeling with Cy3 and FAM fluorophores, respectively. Fluorescent in situ hybridization (FISH) was conducted according to the published method [46]. Approximately 10 μL of hybridization solution containing 1.5 μL FAM-green and 1.5 μL Cy3-red probes was placed onto each treated slide. Subsequently, the hybridization slides were incubated overnight at 37 °C. Coverslips were gently removed and the slides were washed 3 min in 2 × SSC, 10 min in 2 × SSC and 3 min in 1 × PBS, respectively. Slides were dried and counterstained with 4’, 6-diamidino-2-phenylindole (DAPI). FISH images were captured by Olympus BX51 fluorescence microscope.

### Chromatin immunoprecipitation and sequencing (ChIP-seq)

ChIP assays were performed by Shandong Xiuyue Biotechnology Co., Ltd, according to the standard crosslinked ChIP protocol with modifications. The rabbit anti-CENH3 polyclonal antibody used in this study was raised against a chemically synthesized peptide corresponding to the N-terminal 30 amino acids of the *B. napus* ZS11 CENH3 protein (MARTKHFASRARDRNPTNATASSSAAAAAGPS). This region is highly divergent between CENH3 and canonical histone H3, making it a suitable epitope for specific recognition. Briefly, cells were harvested and crosslinked with 1% formaldehyde for 10 min at room temperature. After sonication, immunoprecipitation was performed with Anti-CENH3, N-terminal Antibody (*B. napus*). The immunoprecipitated complex was washed, and DNA was extracted and purified by MinElute PCR Purification Kit (QIAGEN, #28006). The ChIP-seq library was prepared using TruePrep DNA Library Prep Kit V2 for Illumina (Vazyme, #TD501) according to the manufacturer’s instructions. For ChIP-seq, extracted DNA was ligated to specific adaptors followed by deep sequencing in the Illumina Novaseq 6000 using 150 bp paired-end.

### ChIP-seq data alignment and processing

Sequencing reads were quality-controlled and filtered using Fastp (v1.0.1) [47] with default parameters. Filtered ChIP and Input reads were aligned to the reference genome using Bowtie2 (v2.5.4) [48] with parameters “--very-sensitive --no-mixed --no-discordant -k 10”, following previous *Arabidopsis* studies [10]. Normalized ChIP/Input values were calculated using bamCompare from DeepTools (v3.5.6) [49] with the parameters “--binSize 1000 -p 72 --effectiveGenomeSize 1017632034 --normalizeUsing BPM --operation log2 --scaleFactorsMethod None --outFileFormat bedgraph”. CENH3 ChIP–seq peaks were identified genome-wide using the callpeak module of MACS2 (v2.1.1) [50]. The following parameters were applied: --keep-dup all -g effective_genome_size -n sample -f BAM -B --nomodel –shift −100 --extsize 200 --outdir peak --nolambda. Among these, “--keep-du all” was used to retain all duplicate reads, thereby minimizing potential bias introduced by PCR amplification preferences.

### Karyotype evolution

WGDI [51] was used to infer the ancestral karyotype composition of *B. napus* chromosomes based on previously published *Brassica* ancestral karyotype data [52] with parameters “score = 100, evalue = 1e-5, repeat_number = 5”. Synteny between *B. napus* chromosomes and ancestral chromosomes was visualized using MCScan (Python version) [53].

### LTR insertion time and removal efficiency

LTR insertion times were estimated using LTR_retriever (v2.9.0) [54] with default parameters. The script solo_intact_ratio_wrap.sh from this package was used to calculate the ratio of solo-LTRs to intact LTRs in centromeres of different *Brassica* species. Statistical analyses and corresponding plots were generated using the R package ggpubr (https://github.com/kassambara/ggpubr).

## Supplementary Information

Additional file 1: Supplementary Figures. Supplementary Figures S1-S24.

Additional file 2: Supplementary Tables. Supplementary Tables S1-S13.

## Data Availability

The raw sequencing data generated in this study have been deposited in the NCBI Sequence Read Archive (SRA) under BioProject accession PRJNA1398648 and SRA study accession SRP659942 [55], including PacBio HiFi reads (SRR36686106), Oxford Nanopore ultra-long reads (SRR36686105), and CENH3 ChIP-seq and input libraries (SRR36686103 and SRR36686104, respectively). The ZS11RS3 genome assembly has been deposited in GenBank under accession number JBWBZS000000000.1, associated with the same BioProject [55]. Previously published genome sequencing and Hi-C datasets for B. napus ZS11 used in this study were retrieved from NCBI BioProject PRJNA546246 [22, 56]. The ZS11RS3 genome assembly generated in this study are archived in NCBI GenBank and SRA as the primary public repositories and are also available through the Brassica Information Gateway (BIGW; https://brassica.swu.edu.cn/bigw/), which serves as a public portal for genome download and interactive visualization. Specifically, the BIGW Download module provides access to the ZS11RS3 genome assembly and annotation files, while the T2T Genome module enables interactive visualization of the telomere-to-telomere reference genome and associated gene models. All scripts used in this study are publicly available on GitHub (https://github.com/Xin-DongXu/ZS11RS3) [57], and a permanent archived version has been deposited in Zenodo (https://doi.org/10.5281/zenodo.19631892) [58]. The GitHub repository is distributed under the MIT License.
