# Supplementary material for: A complete reference genome reveals genetic landscape of Brassica centromeres
Source: Genome Biol. 2026 Jul 27;27:333. doi: 10.1186/s13059-026-04218-4 (PMC13721389; doi:10.1186/s13059-026-04218-4)
Supplement: Supplementary file 1 [file 13059_2026_4218_MOESM1_ESM.docx]

**Fig. S1. Genome-wide distribution of previously unassembled regions (PURs) across chromosomes in the ZS11RS3 assembly.** PURs are mapped along each chromosome in the ZS11RS3 genome. Salmon-colored blocks indicate PURs, with block lengths proportional to the corresponding region sizes.

**Fig. S2. Synteny between the *B. napus* ZS11RS3 genome and the X5A genome.** Genome-wide collinearity between the ZS11RS3 (upper panel, blue) and X5A (lower panel, green) assemblies is illustrated using pairwise sequence alignments. Chromosomes from the A and C subgenomes are labeled along each genome (A01–A10 and C01–C09). Colored links between the two genomes represent different types of structural relationships: syntenic regions (light gray), inversions (orange), and translocations (yellow).

**Fig. S3. Sequence features of the *B. napus* ZS11RS3 centromeres.** a, Circos plot showing satellite repeat annotations across the ZS11RS3 genome assembly. Each repeat is color-coded according to its monomer length (bp). The accompanying histogram displays the copy number distribution of satellite repeat monomers of different lengths. b, Frequency distribution of *BnaCEN176* sequence variants across the consensus repeat in the A and C subgenomes. The DNA sequence below is colour-coded by nucleotide type. c, Dot plots comparing the 19 functional centromeres of the *B. napus* ZS11RS3 genome using search windows of 120 bp and 178 bp. Red and blue indicate sequence similarity on the forward and reverse strands, respectively.

**Fig. S4. Identification of satellite DNA sequences in the *B. napus* ZS11 v0 genome.** Circos plot showing satellite repeat annotations across the ZS11 v0 genome assembly. Each repeat is color-coded according to its monomer length (bp). The accompanying histogram displays the copy number distribution of satellite repeat monomers of different lengths.

**Fig. S5. Histogram of CENH3 ChIP–seq peak distribution across the ZS11RS3 genome.** Distribution of CENH3 ChIP–seq peaks along the ZS11RS3 genome. The red color gradient indicates the number of peaks, with darker shades representing higher peak densities.

**Fig. S6. Multiple sequence alignment of the five newly identified satellite monomers with *BnaCEN176*.**

**Fig. S7. Genome-wide distribution of five newly identified centromeric satellite monomers.** Genome-wide distribution profiles of TRF_00793 (a), TRF_01213 (b), TRF_03610 (c), TRF_09081 (d), and TRF_12872 (e) across all chromosomes of *B. napus* ZS11RS3.

**Fig. S8: The *B. napus* ZS11 CEN176 satellite repeat library.** a, Histograms of the distance between HORs, *BnaCEN176* monomer lengths, mean number of variations per monomer, and *BnaCEN176* HOR lengths (monomers). b, Plot of the distance between pairs of *BnaCEN176* HORs and divergence.

**Fig. S9. CENH3 ChIP–seq signal and the distribution of LTRs and satellite repeats across the Centromere Dip Regions (CDRs) of chromosomes A01–A10 and C01–C09.** From top to bottom, the three tracks show the CENH3 ChIP–seq signal, the proportion of LTR elements per 1-kb window, and the proportion of satellite sequences per 1-kb window, respectively.

**Fig. S10. Subgenome-specific differences in centromere length and LTR insertion dynamics in *B. napus*.** a, Chromosome lengths of the *B. napus* A subgenome (BnA) and *B. napus* C subgenome (BnC), shown as boxplots. Statistical significance was assessed using a t-test; asterisks indicate significant differences between groups (**P* < 0.05, ***P* < 0.01, ****P* < 0.001). b, Centromere lengths of each chromosome in the BnA and BnC, shown as boxplots. c, Histograms and boxplots of LTR insertion times inside (red) versus outside (blue) centromeres in the *B. napus* ZS11RS3 genome. d, Histograms and boxplots of LTR insertion times in BnA and BnC centromeres.

**Fig. S11. The structural organization and compositional characteristics of the A02-A10 centromeres.** From top to bottom, the tracks represent gene density, LTR/*Gypsy* density, LTR/*Copia* density, chromosomal distribution of rDNA loci, copy number of *BnaCEN176* calculated in 10-kb windows (red indicates the forward strand and blue indicates the reverse strand), CENH3 enrichment shown as log2(ChIP/Input) values (red indicates values greater than zero and blue indicates values less than zero), a schematic representation of the chromosome with the centromeric region highlighted in black, the Hi-C contact heatmap of chromosomes with orange triangles indicating TADs.

**Fig. S12. The structural organization and compositional characteristics of the *B. napus* ZS11 C subgenome.** From top to bottom, the tracks represent gene density, LTR/*Gypsy* density, LTR*/Copia* density, chromosomal distribution of rDNA, copy number of *BnaCEN176* calculated in 10-kb windows (red indicates the forward strand and blue indicates the reverse strand), CENH3 C enrichment shown as log2(ChIP/Input) values (red indicates values greater than zero and blue indicates values less than zero), a schematic representation of the chromosome with the centromeric region highlighted in black, the Hi-C contact heatmap of chromosomes with orange triangles indicating TADs.

**Fig. S13. Statistical and comparative analysis of TADs in the genome of *B. napus* ZS11RS3.** a, Comparison of TAD sizes inside and outside the centromere. b, Comparison of the minimum distance between centromeric boundaries of *B. napus* A/C subgenomes (BnA/BnC) and TAD boundaries.

**Fig. S14. Statistical and comparative analysis of CENH3 ChIP-Seq enrichment levels in chromosomal regions containing rDNA-enriched pericentromeres (REPs).** Panels a-d show the comparison of CENH3 log2(ChIP/Input) values across different regions of *B. napus* chromosomes A01, A05, A06, and A09, respectively.

**Fig. S15. Distribution of rDNA across the genomes of *Brassica* species and *Cardamine hirsuta*.** Grey rectangles represent chromosomes, and red blocks are scaled proportionally according to the length of the rDNA.

**Fig. S16. Evolutionary dynamics of the karyotypes of chromosomes A01, A06, and A09 in ZS11, from the ancestral ACBK.** From top to bottom: syntenic relationships between ACBK and each of A01, A06, and A09; distribution of rDNA along each chromosome, with different colors representing distinct rDNA types; ACBK-derived composition of A01, A06, and A09 inferred by WGDI.

**Fig. S17. Comparative analysis of centromeric TE composition and LTR turnover in *Brassica*.** a, Proportions of different TE types and satellite monomers detected inside (bottom) or outside (top) *BnaCEN176* arrays. Bin size = 1 Mb; the height of the bars below represents LTR insertion time, with LTRs of insertion time = 0 labeled directly on the chromosomes. b, LTR insertion times within centromeres of each *B. napus* ZS11RS3 chromosome. c, Boxplots of solo-to-intact LTR ratios in centromeres across *Brassica* species.

**Fig. S18. Distribution of transposable element classes and satellite monomers within and flanking centromeres in the A subgenome of *B. napus*.** Proportions of different transposable element (TE) classes and satellite monomers detected within (bottom) and outside (top) the *BnaCEN176* arrays. Data are summarized in 1-Mb bins.

**Fig. S19. Distribution of transposable element classes and satellite monomers within and flanking centromeres in the C subgenome of *B. napus*.** Proportions of different transposable element (TE) classes and satellite monomers detected within (bottom) and outside (top) the *BnaCEN176* arrays. Data are summarized in 1-Mb bins.

**Fig. S20. Distribution of transposable element classes and satellite monomers within and flanking centromeres in the *B. rapa* genome.** Proportions of different transposable element (TE) classes and satellite monomers detected within (bottom) and outside (top) the *BnaCEN176* arrays. Data are summarized in 1-Mb bins.

**Fig. S21. Distribution of transposable element classes and satellite monomers within and flanking centromeres in *B. oleracea* genome.** Proportions of different transposable element classes and satellite monomers detected within (bottom) and outside (top) the *BnaCEN176* arrays. Data are summarized in 1-Mb bins.

**Fig. S22. Temporal and spatial distribution of LTR insertions across the ZS11RS3 genome.** a, LTR positions are indicated by lines, colored according to the superfamily to which the LTR belongs. The length of each line represents the insertion time (Mya). For recent LTR insertions with an insertion time of 0, the lines are directly annotated on the chromosomes. b, Comparison of LTR insertion times among rDNA-enriched pericentromeric regions.

**Fig. S23. Statistical analysis of centromeric sequence composition across *Brassica* subgenomes.** Pie charts show the proportional contributions of different sequence components within the centromeres of each chromosome across *Brassica* species, with major categories including LTR/*Gypsy*, LTR*/Copia*, LTR/unknown, and satellite repeats.

**Fig. S24. Distribution of transposable element classes and satellite monomers within and flanking centromeres in the *Arabidopsis* genome.** Proportions of different transposable element (TE) classes and satellite monomers detected within (bottom) and outside (top) the *AthCEN178* arrays. Data are summarized in 1-Mb bins.
